# Nematic Liquid-Crystal Colloids

**DOI:** 10.3390/ma11010024

**Published:** 2017-12-25

**Authors:** Igor Muševič

**Affiliations:** 1 J. Stefan Institute, Jamova 39, Ljubljana SI-1000, Slovenia; igor.musevic@ijs.si; 2 Faculty of Mathematics and Physics, University of Ljubljana, Jadranska 19, Ljubljana SI-1000, Slovenia

**Keywords:** liquid crystal, colloids, experimental topology, handlebodies

## Abstract

This article provides a concise review of a new state of colloidal matter called nematic liquid-crystal colloids. These colloids are obtained by dispersing microparticles of different shapes in a nematic liquid crystal that acts as a solvent for the dispersed particles. The microparticles induce a local deformation of the liquid crystal, which then generates topological defects and long-range forces between the neighboring particles. The colloidal forces in nematic colloids are much stronger than the forces in ordinary colloids in isotropic solvents, exceeding thousands of *k_B_T* per micrometer-sized particle. Of special interest are the topological defects in nematic colloids, which appear in many fascinating forms, such as singular points, closed loops, multitudes of interlinked and knotted loops or soliton-like structures. The richness of the topological phenomena and the possibility to design and control topological defects with laser tweezers make colloids in nematic liquid crystals an excellent playground for testing the basic theorems of topology.

## 1. Introduction

**Figure 1:**
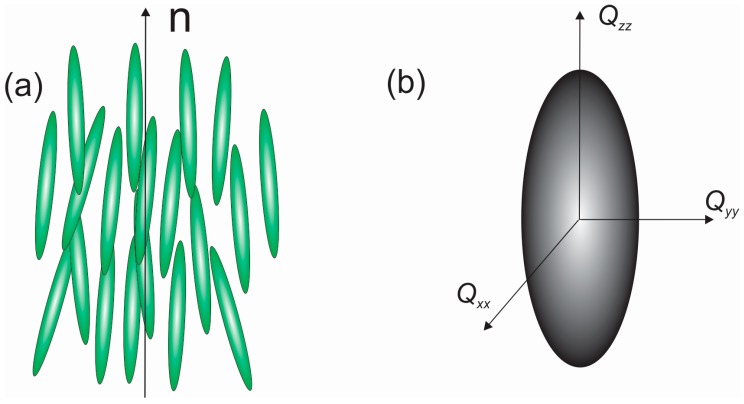
Molecular ordering and tensor material properties of a nematic liquid crystal: (**a**) Snapshot of molecular order in a nematic liquid crystal. Molecules exhibit rapid diffusion and orientational fluctuations; (**b**) Orientational order is described by the *Q*-tensor, where the order parameter *S* (a number) is the largest eigenvalue of this tensor.

In a nematic liquid crystal, rod-like organic molecules are on average spontaneously oriented along the direction called the director **n**, as shown in Figure 1. The director can have an arbitrary direction in space, but in reality it always points along some preferred direction, because the liquid crystal is usually confined to a measuring cell. The degree of order of the nematic liquid crystal is described by the order parameter *S* [1],

$$S=\frac{1}{2}\langle \left(3\;\cos^{2}\theta \;-\;1\right)\rangle$$

Here, *θ* is the angle between the long axis of a selected molecule and the average direction of the orientation, the director **n**. The brackets 〈〉 denote the average over the angular distribution of molecules in the sample. The order parameter is temperature dependent and decreases when the temperature of the nematic liquid crystal is increased. If the nematic liquid crystal is heated above a certain temperature, the orientational order is suddenly and completely lost and there is a first-order phase transition into the isotropic state of the nematic liquid crystal. In this isotropic phase, the nematic liquid crystal behaves as an ordinary liquid with complete orientational disorder, and therefore S=0. It should be noted that the nematic state of matter is positionally disordered; hence, it is similar to positionally disordered fluids.

Because of the spontaneous orientational order of the nematic liquid crystal, the material properties of this state of matter are strongly anisotropic. For example, nematic liquid crystals have the largest optical birefringence Δn observed in matter, which typically ranges from 0.1 to 0.4. The electric and magnetic properties of nematic liquid crystals are also anisotropic. The dielectric constant, for example, is a tensorial property with very different eigenvalues, typically between ε 10 and 1. The magnetic properties of liquid crystals are similar to those of anisotropic diamagnetic materials, and other physical properties, such as the electric conductivity and viscosity, are anisotropic as well.

The appearance of spontaneous order is not only reflected in the anisotropy of the physical properties, but also in the elasticity of this state of matter. Nematic liquid crystals are fluids because they can flow, but they also exhibit orientational elasticity and are able to transmit static torques. The elastic properties of liquid crystals are used in display applications, where the elasticity restores the original state after the external electric field is switched off, therefore driving the pixel element into the off state. 

Nematic liquid crystals can also be aligned microscopically by putting them in contact with certain surfaces. For example, when a nematic liquid crystal is put into contact with a solid crystal, such as graphite, the molecules are forced to lie flat along the interface throughout the contact region. This type of alignment is called planar alignment. Other types of surfaces, for example, hairy polymer surfaces, are able to induce either planar or perpendicular alignment anchoring of the liquid crystal at their interface.

The combination of the long-range orientational order and the ability to align the director field along the surface is the key characteristic of nematic liquid-crystal colloids, also short-named as nematic colloids [2]. Here, micrometer-sized particles, such as microspheres, are immersed in the nematic liquid crystal. Because of the surface alignment, the director field is forced to align along a closed surface of the sphere, which results in the appearance of topological defects of the nematic orientational field [3,4,5,6]. Topological defects are regions where the order cannot be defined and, as a consequence, the orientational order is strongly depressed in the core of topological defects. This creates an interesting situation where the colloidal inclusions are inevitably accompanied by topological defects and the liquid crystal surrounding the colloidal particle is strongly deformed. These deformations result in a strong elastic interaction between neighboring colloidal particles in the nematics. This interaction force triggers the spontaneous assembly of nematic colloids, where the process of pair interaction is strongly characterized by the topological defects. Topological defects are therefore very important for the interaction of nematic colloids and bring in a particular signature related to topology.

When looking through the literature covering a significant time period it is clear that studies of nematic colloids and topological defects appear in waves separated by tens of years. The first studies of defects in nematic liquid crystals were conducted in the 1970s using ordinary optical microscopy [7,8]. The second wave of exploration was triggered by the work of Poulin et al. in 1997 on water droplets in nematics [9], but was limited by the existing experimental methods of that time. During the next ten years, two important experimental techniques were developed and introduced to the study of nematic liquid-crystal colloids: Laser tweezers [10,11,12,13,14,15,16,17,18,19,20,21,22,23,24,25,26,27] and fluorescent confocal polarizing microscopy (FCPM) [28,29,30,31,32]. The first method made it possible to grab and manipulate individual micrometer-sized colloidal particles using laser light focused through the objective of an optical microscope. The forces between the colloidal particles could then be studied and the particles could be systematically assembled into 2D and 3D colloidal crystals. FCPM made it possible to study the director field around the colloidal particles and to explore the richness of the topological defects and the structures around the colloidal inclusions having various levels of complexity.

The aim of this article is to describe the basic concepts and present some results from the field of nematic liquid-crystal colloids to scientists who are outside the field. There is also a short review of the fundamental topological properties of this system, which in my opinion are the most interesting phenomena that are observed in nematic colloids.

## 2. Forces between Spherical Microparticles in 2D Nematic Liquid Crystals

The study of nematic colloids started nearly two decades ago with an experiment performed by Poulin et al. in 1997 [9]. They mixed water and a nematic liquid crystal and obtained a dispersion of micrometer-diameter water droplets floating in the nematic liquid crystal. Under a polarizing optical microscope they saw that the water droplets spontaneously formed chains, which were directed along the nematic director (the direction of the average orientation of liquid-crystal molecules). Although fluid, the water droplets did not coalesce into larger droplets, but remained separated from each other by a topological defect that was spontaneously created in the vicinity of each droplet. This experiment was clear evidence of a new type of force that acts between colloidal inclusions in nematic liquid crystals. When micrometer-diameter colloidal particles are dispersed in the nematic, the strength of this force is typically tens of pN at a typical surface-to-surface colloidal separation of a micrometer or so. The force has its origin in the ordering and elasticity of the nematic liquid crystal and, most importantly, in the alignment of the liquid-crystal molecules along the closed surface of the colloidal inclusion.

**Figure 2:**
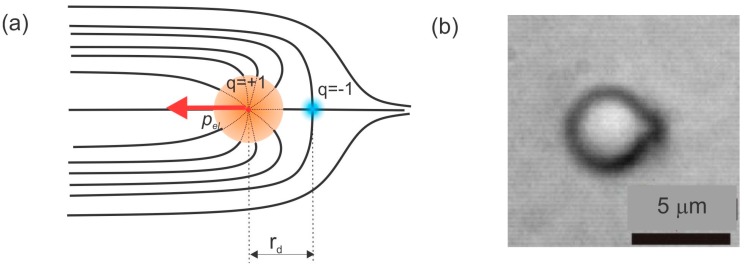
(**a**) A spherical colloidal particle with homeotropic surface anchoring forces the liquid crystal molecules to orient along its closed surface. The director field is represented by black lines. Far away from the particle, the nematic liquid crystal is uniform. However, because of the alignment of the liquid crystal along the closed surface, it is not possible to have a smooth director field everywhere. Instead, regions of disorder are created, where the molecules have no preferential direction to align with. These regions are topological defects. There are two topological defects (blue and red dots), separated by r_d_. The first one is called a hyperbolic hedgehog (blue dot) and carries a topological charge q=−1. The second one is a virtual one and resides in the center of the particle. It is called a radial hedgehog and carries the opposite topological charge q=+1. These two topological defects form an elastic dipole denoted as ${\vec{p}}_{el}$. (**b**) Real image of a dipolar colloidal particle with a dark spot, which is a hyperbolic point defect. No polarizers were used when taking the photograph.

When the colloidal inclusion is introduced into the nematic liquid crystal, the closed surface of the particle forces the liquid-crystal molecules to align all along the closed surface of the inclusion. We shall consider the homeotropic alignment of a liquid crystal on a colloidal surface where the molecules are locally perpendicular to that surface. As shown in Figure 2, it is not possible to smoothly fill the space around such an inclusion with liquid-crystal molecules. There are regions where the liquid-crystal molecules “do not know” in which direction to orient themselves, which results in the local disordering of their long molecular axes. These deformed and disordered regions are called topological defects and are a consequence of the law of the conservation of topological charge.

There are two important aspects of topological defects in liquid crystals. First, the topological defects are regions of reduced orientational order of the liquid-crystal molecules, which are also accompanied by strong elastic deformation of the liquid crystal. Because the liquid crystals are orientationally ordered fluids, their deformation costs energy. Similarly, reducing the degree of local orientational order also costs energy, because the nematic liquid crystal has to be nearly molten to achieve a lower order parameter. Second, in addition to free energy, topological defects are, mathematically speaking, points or loops where the ordering field is singular, i.e., the field is not defined in those regions. The defects of the orientational field are therefore of the same nature as the topological defects in any other physical field. For example, an electric field has its singularities in electric charges, which are the source of this field. In physics, charges always generate forces between themselves, which is also the case with the topological charge attributed to topological defects. In addition, the total topological charge of the system has to be conserved at all times, which means that topological charges are always created in pairs of oppositely charged defects. The point defect accompanying the inserted spherical colloidal particle must be neutralized by another topological charge of opposite sign. In the case of a solid microsphere, this charge is a virtual one and is located in the center of the microsphere, as illustrated in Figure 2. 

There are therefore two oppositely charged topological defects accompanying a microsphere with a homeotropic surface anchoring in the nematic liquid crystal, as shown in Figure 2. The first one is the real topological defect, which has the hyperbolic internal structure of the director field and an axial symmetry around the axis connecting the defect and the center of the sphere. This is called a hyperbolic hedgehog. The second, oppositely charged topological defect has a radial structure and is called a radial hedgehog. Together, they form an elastic dipole ${\vec{p}}_{el}$, because of the strong elastic deformation in the neighborhood of the hedgehogs. It is also called a topological dipole, formed of two oppositely charged point defects.

**Figure 3:**
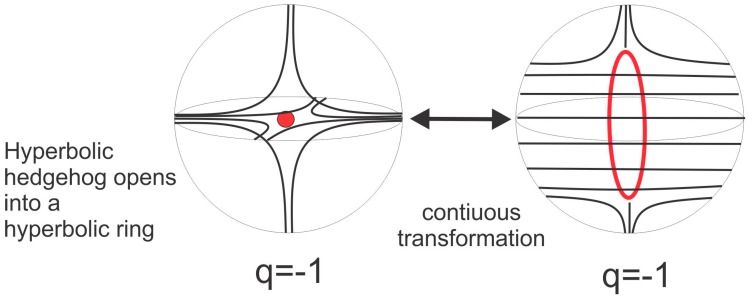
A hyperbolic point can be smoothly opened into a hyperbolic ring, which encircles a real microsphere in a nematic liquid crystal. Adapted from Ref. [2].

**Figure 4:**
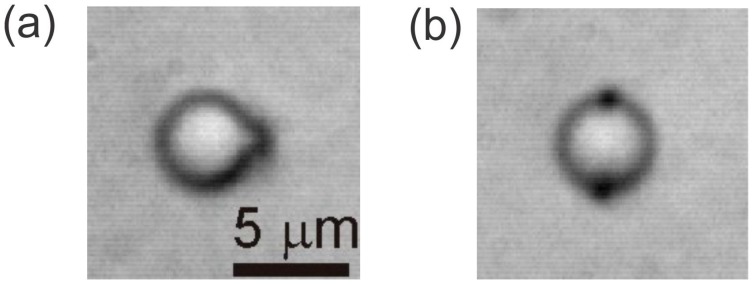
(**a**) A dipolar colloidal particle in a nematic liquid crystal. The dark dot on the right-hand side is a hyperbolic point defect. (**b**) A quadrupolar colloidal particle in the nematic. The two dark spots on the top and the bottom are a cross-section of a hyperbolic ring encircling the microsphere.

In topology, any smooth deformation of objects, such as the stretching of surfaces or the twisting of loops, is allowed. In our case, it is allowable to smoothly open a point into a loop, following the smooth process illustrated in Figure 3 [2,6,33]. This means that a hyperbolic hedgehog could be opened into a hyperbolic ring, and this ring could be smoothly pushed along the surface of the colloidal particle to its equator. In this way, a new type of colloidal particle is formed that has quadrupolar symmetry, as shown in Figure 4b. The hyperbolic ring is also called the Saturn ring [34,35,36,37] because of its similarity to the planet Saturn and its rings. The particle in Figure 4a is a dipolar colloid with a hyperbolic point defect.

Whether a colloidal particle is a dipole or a quadrupole depends on the strength of the orientational surface anchoring and the diameter of the colloidal particle. If the particle is small (less than a micrometer) and the nematic director is forced to follow a strongly curved spherical surface, it will relax the elastic energy of distortion by forming a larger structure, i.e., the Saturn ring. For larger particles and a lower Gaussian curvature, the elastic energy can be stored in a hyperbolic point hedgehog. The dipolar or quadrupolar nature of the particle also depends on the external fields and the confinement. If the particles are confined to a very thin nematic layer, they will usually obtain a quadrupolar structure with a Saturn ring.

**Figure 5:**
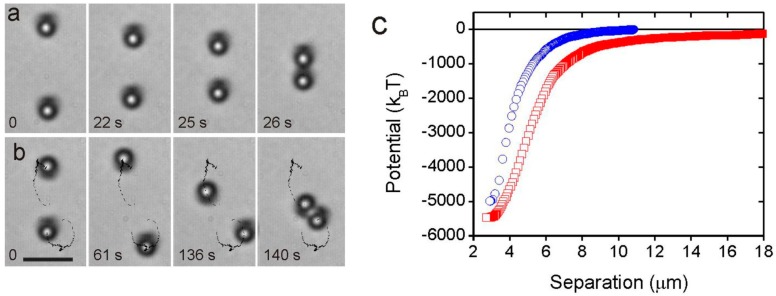
(**a**) Two dipolar particles with the same orientation of their elastic dipoles will attract each other, if placed collinearly. (**b**) If placed side by side, they will first repel each other and find their minimum energy through a side-by-side attraction. The black trail is the trajectory of each particle, as determined by the particle-tracking method. (**c**) Pair potential for collinear attraction shown in (**a**) (squares) and side-by-side attraction in (**b**) (circles). Scale bar, 5 µm. Adapted from Ref. [2].

If two dipolar or quadrupolar particles are brought together, the regions of their elastic distortion start to overlap and the two particles are either attracted to or repelled from each other, as illustrated in Figure 5 for dipolar colloidal particles.

The reason for this pair-interaction force is in the overlapping of the elastically distorted regions and the consequent sharing of the elastic energy. If both elastic fields are in favor of lowering the total free energy, the particles will be attracted to share as much of that energy as possible, which will then reduce the total energy of the pair. If the sharing is energetically unfavorable, the particles will be repelled from each other.

The first studies of the pair-interaction forces between the spherical inclusions in nematic liquid crystals were theoretical and are closely related to studies of the director structure around a spherical inclusion, first analyzed by Terentjev et al. [35,36]. Kuksenok et al. [38] analyzed the Frank free-energy functional provided by the surface-anchoring energy and used multipole expansion for the director field. Ramaswamy et al. [39] predicted the elastic interaction force between colloidal particles that was calculated within an electrostatic analogy following Brochard and de Gennes using multipole expansion [40]. For two quadrupolar particles, they predicted a power-law dependence of the elastic attractive force. A very comprehensive mean-field analysis for the pair interaction of elastic multipoles and in particular topological dipoles was performed by Lubensky et al. [5,9], stressing the importance of the topology. Further mean-field approaches to nematic colloids include investigations of the stability of colloidal clusters by Lev and Tomchuk [41] and the effects of confining walls on the colloidal-pair interaction by Fukuda et al. [42,43]. Fukuda et al. used a fully tensorial Landau-de Gennes approach to the analysis of colloidal-pair interactions in a nematic liquid crystal. Pergamentschik and Uzunova used a refined approach to colloidal nematostatics [44,45,46]. They observed that, in spite of the analogy with electrostatics, the three-dimensional colloidal nematostatics is substantially different in both its mathematical structure and its physical implications. A review of this approach can be found in Ref. [47]. Chernyshuk et al. [48] considered the theory of colloidal elastic interactions in the presence of an external electric or magnetic field using the Green’s function method.

The first experimental evidence for the existence of elastic forces between spherical inclusions in a nematic liquid crystal was given for small-angle neutron scattering (SANS) experiments on a lyotropic nematic crystal by Raghunathan et al. [49]. The first experiments on the pair-interaction forces between inclusions in the nematic liquid crystal were performed in the seminal experiments of Poulin et al. [9]. They performed an experiment on water droplets dispersed in a 5CB nematic liquid crystal. They observed that each water droplet was accompanied by a point defect, and water droplets spontaneously assembled into chains with topological defects separating two water droplets. They explained this chaining of liquid droplets in another liquid by a topological (elastic) dipole-dipole interaction force, which is expected to follow a power law [5,9]. There are many different studies of the elastic forces between spherical colloidal particles in nematics [50,51,52,53,54,55,56,57,58,59,60,61]. The observed forces are relatively strong and long range, and show a power-law dependence on the separation between the two particles, together with a strong angular dependence. They can be well described with an electrostatic analogy and behave like electric dipoles in an external electric field, which is analogous to the far-field nematic director. 

**Figure 6:**
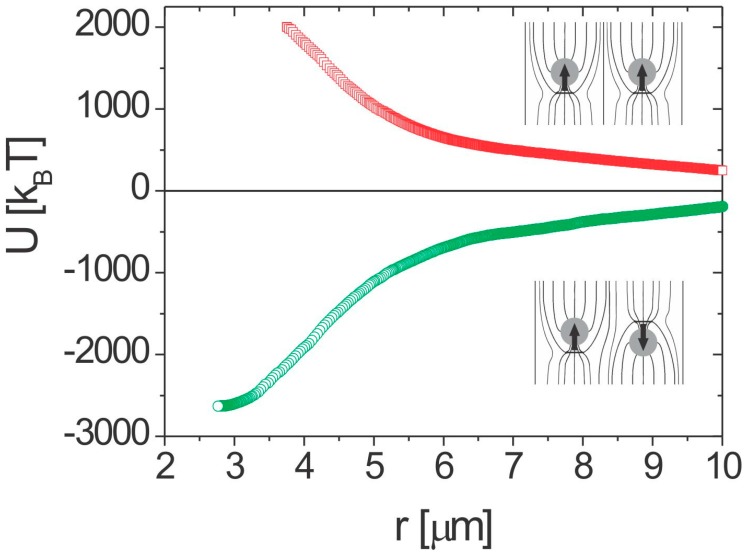
Dipole-dipole pair-interaction energy in nematic liquid crystal for two parallel and antiparallel dipoles. Adapted from Ref. [2].

The elastic colloidal forces can be expressed in terms of multipole moments. Two dipolar particles will therefore show a $1/r^{4}$ interaction force with a strong angular dependence, similar to the electric force between two electric dipoles. It is also possible to measure the pair-interaction energy of the nematic colloids, and it turns out that dipole-dipole binding energies are of the order of 3000 *k_B_T* for 1-micrometer-diameter microspheres. Figure 5c and Figure 6 show the pair-interaction energy for two dipolar colloidal particles, which is obtained by integrating the work of the attractive force along the trajectory of one of the particles. The interaction energies are huge, even for micrometer-size particles, and are due to the energy of the elastically distorted nematic director between and around the colloidal pair.

**Figure 7:**
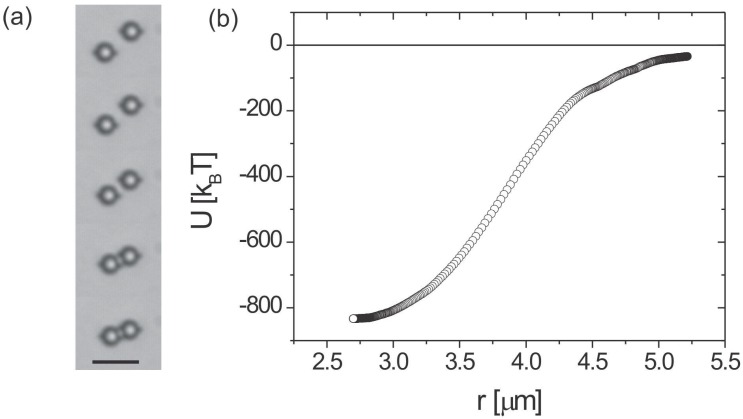
(**a**) Series of snapshots showing the attraction between two quadrupolar nematic colloids. (**b**) Quadrupole-quadrupole pair-interaction energy in a nematic liquid crystal. Scale bar, 5 µm. Adapted from Ref. [2].

Two quadrupoles show a $1/r^{6}$ interaction force, which is much weaker than the dipole-dipole force. An example of the measured pair-interaction energy for two quadrupolar colloidal particles is shown in Figure 7. Typically, the quadrupolar colloidal interaction is several times weaker than the dipolar one for the same size of particles.

Finally, the dipole-quadrupole interactions show a $1/r^{5}$ power-law dependence, similar to the interaction of an electric dipole with an electric quadrupole. Because of the power-law interaction nature of this interaction force, two micrometer-diameter colloidal particles in the nematic will interact across a very large separation of tens of micrometers.

## 3. Microrods in Nematic Liquid Crystals

**Figure 8:**
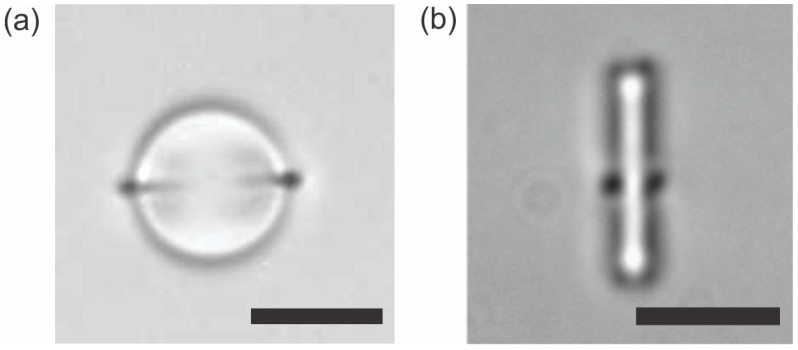
(**a**) The Saturn ring is encircling a microsphere with homeotropic surface anchoring in a nematic liquid crystal. (**b**) The Saturn ring is also encircling a microrod with homeotropic surface anchoring. Scale bars, 10 µm.

The force of the interaction in nematic liquid-crystal colloids has a strong topological signature, because of the presence of topological defects. The interaction force is strongly anisotropic, because the appearance of the topological defect breaks the continuous rotational symmetry of a spherical colloidal particle. However, it is allowed in topology to smoothly deform objects and this smooth “morphing” of the object by stretching and deforming the surface will keep their topological properties unchanged. This striking property is reflected and observed in real experiments with colloidal particles of different shapes in nematic liquid crystals [62,63,64,65,66,67,68,69,70]. For example, we can smoothly transform a sphere into a short cylinder by stretching and morphing the surface of the sphere. In terms of topology, the cylinder will have the same sort of topological defects as the sphere, which is indeed observed in the experiments [63], as shown in Figure 8.

Figure 8 shows a microsphere and a microrod, both being encircled by a Saturn ring. This is a clear manifestation of the strength of the topology in nematic colloids, suggesting that a microsphere and a microrod are topologically equivalent. Indeed, the nature of the topological defects that are accompanying the objects inserted in a nematic liquid crystal is governed by the Euler characteristics of the object or, alternatively, the genus g of that object [2]. Loosely speaking, genus denotes the number of handles of the object. For example, a sphere has no handles and the genus of the sphere is g = 0. By attaching a single handle to the sphere we obtain an object that is homotopic to a toroid. The toroid therefore has genus g = 1. By increasing the number of handles, the genus of the object increases in proportion to the number of handles. 

The appearance of topological defects in a nematic liquid crystal around an immersed particle is governed by the basic laws of topology and will be explained in more detail in Section 6. Briefly, a topological charge is attributed to the topological defects and similar to Gauss’s law in classical electrostatics this charge is measured by embracing the defect with a closed surface and measuring the number of streamlines of the director field through that surface. If a vector field is pinned to orient perpendicular to a closed surface, the Gauss-Bonnet theorem states that the topological charge of the surface is connected to the Euler characteristic of that surface. In our case, this surface is the surface of the object that is immersed in the liquid crystal. Because the overall topological charge of the inserted object and the liquid crystal around that object must be conserved at all times, the total topological charge is zero. The topological charge in this case consists of the topological charge of the surface and the topological charge of defects accompanying that surface. This means that the topological charge of the defects in a liquid crystal is directly related to the Euler characteristics of the surface of the inserted object. For a microsphere with genus g = 0, the Euler characteristics is 1 − g and there should be a unit topological charge accompanying the inserted spherical surface. In our case this unit topological charge is a hyperbolic hedgehog or a hyperbolic Saturn ring. Because the rod is homotopic to a sphere and has genus g = 0, it should also be accompanied by a unit topological charge, i.e., a hyperbolic hedgehog or a hyperbolic Saturn ring, as observed in the experiments. 

The force between two microrods in nematic liquid crystals is therefore expected to be similar to the force between two microspheres in nematic liquid crystals, with possible variations due to the different geometry (shape) of a rod compared to a sphere. Experimental studies of the pair-interaction forces between microrods in a nematic liquid crystal indeed confirm the predicted separation dependence of the interaction forces [63].

**Figure 9:**
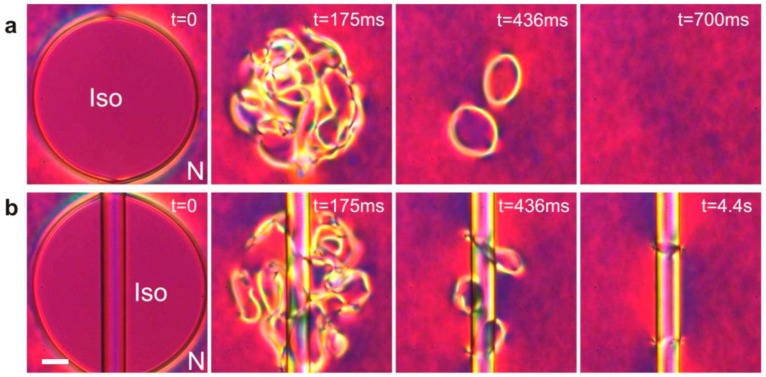
(**a**) Laser-tweezers-induced quench of a nematic liquid crystal without a fiber creates a tangle of topological defects, which all annihilate back to the vacuum. (**b**) If a long fiber is present, most of the defects annihilate, except for a pair of rings encircling the fiber. These two rings are like a particle and its anti-particle carrying opposite topological charges. Scale bar, 10 µm. Adapted from Ref. [71].

Whereas topology determines the unit charge of a defect accompanying a microrod, the number of defects on such a rod is not limited, as long as the total topological charge of all the defects adds up to −1. This could be realized on a very long fiber that is immersed in a nematic liquid crystal, where the separation between the defects should be large enough to prevent the spontaneous attraction and annihilation of the oppositely charged defects. The question of how to create additional defects on a fiber (or any other object) was solved by Nikkhou et al. [71,72,73,74], who used laser tweezers to heat and quench the micrometer-diameter area of a nematic liquid crystal surrounding the microfiber. By using a relatively strong laser light, the liquid crystal is locally heated into the isotropic phase, which creates a circular island of molten liquid crystal surrounded by a nematic liquid crystal. If such an experiment is performed using a thin layer of nematic liquid crystal without a fiber, after the light is switched off, we observe from a sequence of images shown in Figure 9a dense tangle of topological defects. At the beginning of the quench, the structure of this quenched region is finely grained, with the grains representing individual domains of the nematic liquid crystal, which are randomly oriented. These domains are growing with time and they meet each other, forming topological defect structures at their interfaces. 

This phenomenon, well known in cosmology as the Kibble mechanism of monopole production [75,76,77,78], involves a field being rapidly quenched across a phase transition. This results in a number of topological defects, which are regions where various domains of nucleated low-temperature phase meet. In condensed matter this mechanism was translated by Zurek and is one of the current hot topics in the physics of phase transitions. At later stages of the Kibble-Zurek mechanism the topological defects annihilate, resulting in a coarsening of the structure [77,78]. In our case, the final state is a vacuum if there is no fiber present in the nematic liquid crystal. 

The presence of the fiber alters the outcome of the coarsening dynamics after the thermal quench, as seen from the sequence of images in Figure 9b. Instead of a complete annihilation of all the topological defects, a pair of topological defects remains after the coarsening dynamics. If they are far apart, these two rings are stable objects; if they are brought close to each other using the laser tweezers, they start attracting each other and rapidly annihilate to vacuum. This is clear evidence that these two rings are analogous to a particle and its anti-particle. They both carry a unit topological charge of opposite polarity and their total topological charge adds up to zero. In experiments with stronger laser light and a larger starting isotropic area, several pairs of topological defects could be created. Topological defects always appear in an even number, so that their total topological charge is kept to zero in all experiments. There are many different ways of producing oppositely charged pairs of topological charges on a fiber, depending on the orientation of the fiber with respect to the nematic director, the thickness of the cell and the chirality of the liquid crystal. A nematic liquid crystal with a fiber that is a strongly elongated colloidal particle is in fact an ideal experimental setting for studying the topology of the director field with inclusions. 

## 4. Assembly of 2D and 3D Nematic Colloidal Crystals

**Figure 10:**
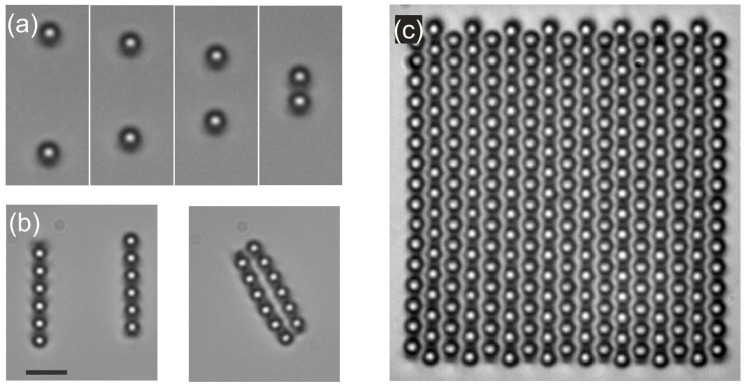
The 2D dipolar colloidal crystal is assembled in several steps by using the laser tweezers to grab and manipulate the colloidal particles. (**a**) The dipolar colloidal particles are pairwise assembled into dipolar colloidal chains. (**b**) Individual dipolar chains are assembled into small 2D colloidal crystallites. (**c**) Additional dipolar chains are brought close to the already assembled structures so that they spontaneously assemble into large 2D colloidal crystals. Scale bar, 5 µm. Adapted from Ref. [2].

The pair-wise attraction of colloidal particles in nematics is very strong and long range, and can therefore be used to assemble 2D and even 3D colloidal crystals. In 2D the colloidal crystals of dipolar, quadrupolar and mixed (dipole-quadrupole) structures were demonstrated. An example of building up a dipolar 2D colloidal crystal is shown in Figure 10 [55,79]. Isolated dipolar colloidal particles are first brought together to assemble into dipolar colloidal chains, as shown in Figure 10a. The orientation of the dipolar chain could be controlled by choosing the proper direction of the individual dipolar particles forming the chain. The direction of the topological dipole could also be changed by using laser tweezers [21]. After a chain is formed, another chain is assembled in which the dipoles are pointing in the opposite direction with respect to the first chain (Figure 10b). When these two chains with anti-parallel dipoles are placed close to each other, they attract and form a small 2D crystallite. This procedure is then repeated by adding dipolar chains with opposite directions of dipoles, which results in rather large 2D dipolar colloidal crystals, as shown in Figure 10c.

A 2D colloidal crystal consists of micrometer-diameter colloidal particles that are very strongly bound together, which makes them very robust, but still relatively elastic. The space between the microsphere is filled with an elastically distorted liquid crystal, which acts as a continuum elastic medium binding the microspheres together. The lattice spacing of a 2D nematic colloidal crystal can be controlled by an external electric field. Due to the dielectric anisotropy, the liquid-crystal molecules in between the microspheres tend to align either along or perpendicular to the field, which forces the microsphere to move apart or closer together. In this way, the lattice constant of a dipolar 2D colloidal crystal could be changed by up to 10%.

Like with dipolar colloids, quadrupolar colloids also form 2D colloidal crystals [60,79]. The difference is in the much weaker binding of the quadrupolar colloidal crystal with respect to the dipolar colloidal crystal, as the binding energies are an order of magnitude smaller. It is also possible to assemble 2D colloidal structures from dipolar and quadrupolar colloidal particles [61,80,81]. This mixed interaction is very interesting [61], because it is strongly anisotropic and shows several possible stable constellations of a colloidal pair. As a result, the variety of dipolar-quadrupolar 2D colloidal crystals is very large, with a huge number of different motifs [81] that can be achieved by laser-tweezers-assisted assembly.

**Figure 11:**
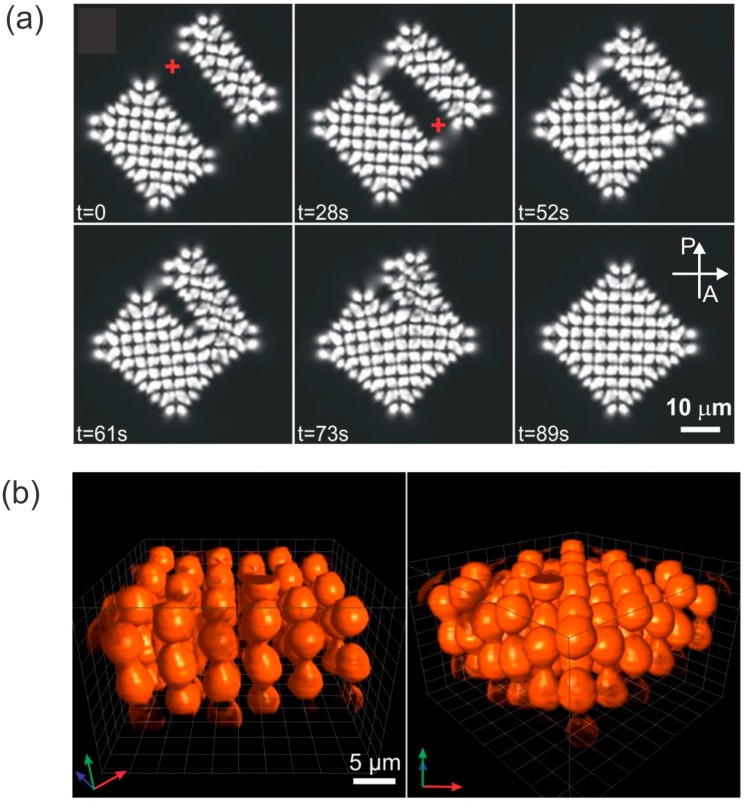
(**a**) Snapshots of the assembly process for a 3D nematic colloidal crystal and (**b**) 3D reconstruction using fluorescent confocal microscopy. P and A indicate the direction of the polarizer and analyzer, respectively. Adapted from Ref. [82].

Whereas the assembly of nematic colloids in 2D thin layers is relatively straightforward, it is very difficult to control a colloidal assembly in 3D. This is because of the difficulty involved in a 3D observation of colloidal structures in optically birefringent liquid crystals and the difficulty of manipulating the colloidal particles in 3D using laser tweezers. In spite of these difficulties, Nych et al. [82] demonstrated the successful assembly of dipolar nematic colloids in 3D nematic colloidal crystal, as shown in Figure 11. The structure is tetragonal with anti-parallel dipolar colloidal chains regularly distributed in space. Similar to 2D nematic colloidal crystals, the 3D nematic colloidal crystals respond strongly to an external electric field. Using positive-dielectric-anisotropy liquid crystal, the crystal can shrink by up to 30%, whereas in a negative-dielectric-anisotropy liquid crystal, the 3D crystal can be rotated by tens of degrees.

## 5. Entanglement, Knotting and Linking of Nematic Colloids in 2D

**Figure 12:**
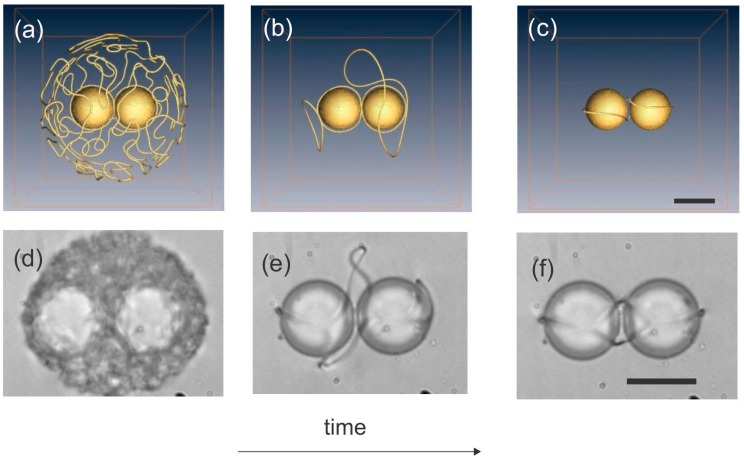
(**a**–**c**) Numerical experiment where the liquid crystal is rapidly cooled across the isotropic-nematic phase transition, leaving behind a single defect loop entangling a pair of colloidal particles. Scale bar 1 µm. (**d**–**f**) In real experiments, rapid quenching across the isotropic-nematic phase transition is performed by using laser tweezers. Scale bar, 20 µm. Numerical panels courtesy of M. Ravnik.

Single colloidal particles with a simple shape, such as a sphere or a short rod, are accompanied by a single topological defect in the form of a point or a small loop. The numerical simulations of different groups in 2003 and 2006 predicted the existence of a new colloidal state, where two or many colloidal particles are entangled together by a single disclination loop [83,84,85,86]. Figure 12 shows the prediction of Landau-de Gennes numerical modeling [86] for the rapid cooling of a nematic liquid crystal around two colloidal particles. At the beginning, a dense tangle of topological defects is formed around both colloidal particles, which gradually annihilate until a single loop is left, as shown in the final panel of Figure 12.

In real experiments [86] the rapid quenching of the nematic liquid crystal with colloidal particles is obtained by using high-power laser tweezers. The tweezers are focused to a particular place in the sample close to the dispersed particles and the optical power is increased to the desired level. Because of the light absorption in a liquid crystal or in glass slides covered with a thin transparent layer of Indium Tin Oxide (ITO), the liquid crystal is locally molten into the isotropic phase, as shown in Figure 12d. Similar to a numerical experiment, the real experiment shows the rapid annihilation of topological defects until a single loop is left, which entangles both the colloidal particles. 

**Figure 13:**
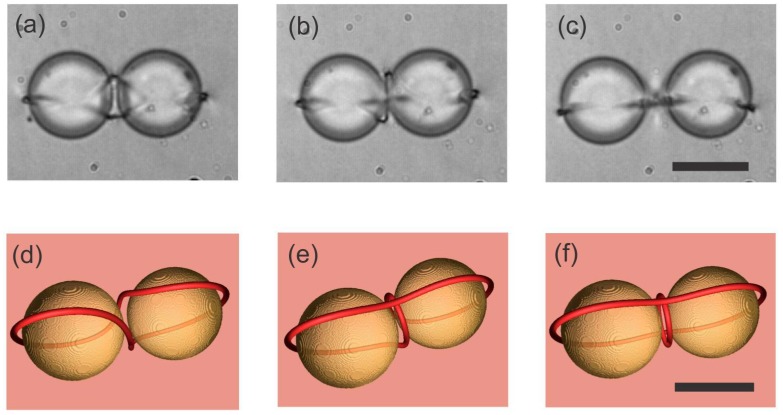
Entangled colloidal pairs appear in three different configurations. (**a**) Figure of 8, observed in the experiment, and (**d**) LdG simulation. (**b**) Figure of Omega in the experiment and (**e**) LdG simulations. (**c**) Entangled point defect in the experiments and (**f**) theory. Scale bar in (**a**–**c**), 20 µm. Scale bar in (**d**–**f**), 1 µm. Adapted from Ref. [86].

There are three different configurations of the defect loop entangling two colloidal particles, which appear in real experiments with different probabilities of their formation, as shown in Figure 13 [86]. The first configuration is the figure-of-eight entanglement, shown in Figure 13a, whereas the simulation is shown in Figure 13d. Here, the −1/2 defect loop is entangling both colloidal particles in a form resembling the number 8. This configuration is most probable and is formed in approximately 36% of experiments [86]. The second most probable configuration is the figure of Omega, shown in Figure 13b,e. It is a single loop that does not cross itself, as in case of the figure of eight, but makes a small loop in between the two colloidal particles. The least probable and also thermodynamically unstable configuration is the entangled point defect, where an extra point defect is located in between two colloidal particles and the −1/2 loop is entangling them, as shown in Figure 13c,f.

The −1/2 defect loop, which appears in all three configurations of entanglement, is an object that requires energy in order to form. In the first-order approximation, this energy is simply proportional to the physical length of the loop. This means that the loop acts as a kind of string that provides constant force. If the two colloidal particles are forced to move apart by an external force, a constant force is needed to separate the colloids. The string-like force is therefore similar to the force of the surface tension. It is possible to measure this force by using two optical traps that grab each of the particles and separate them. If the light is switched off, the two colloidal particles will be driven back by the force of the entanglement. By using a standard technique of particle tracking with video microscopy, it is possible to determine the force between the particles and the work of this force during the motion of the colloids. It turns out that the force of entanglement is even larger than the colloidal forces due to the sharing of defects and the elastic distortion. Typically, the two colloidal particles are entangled by an energy close to 10,000 *k_B_T*. When the experiments with the colloidal entanglement are performed in planar nematic cells where the liquid crystal is homogeneous, it is possible to entangle many colloidal particles into chains or “colloidal wires”. However, it is not possible to entangle colloidal particles in 2D to form an entangled colloidal crystal, although such structures were predicted by numerical simulations. The reason for this is the difficulty of propagating a −1/2 defect line along an arbitrary direction in a homogeneous nematic liquid crystal.

**Figure 14:**
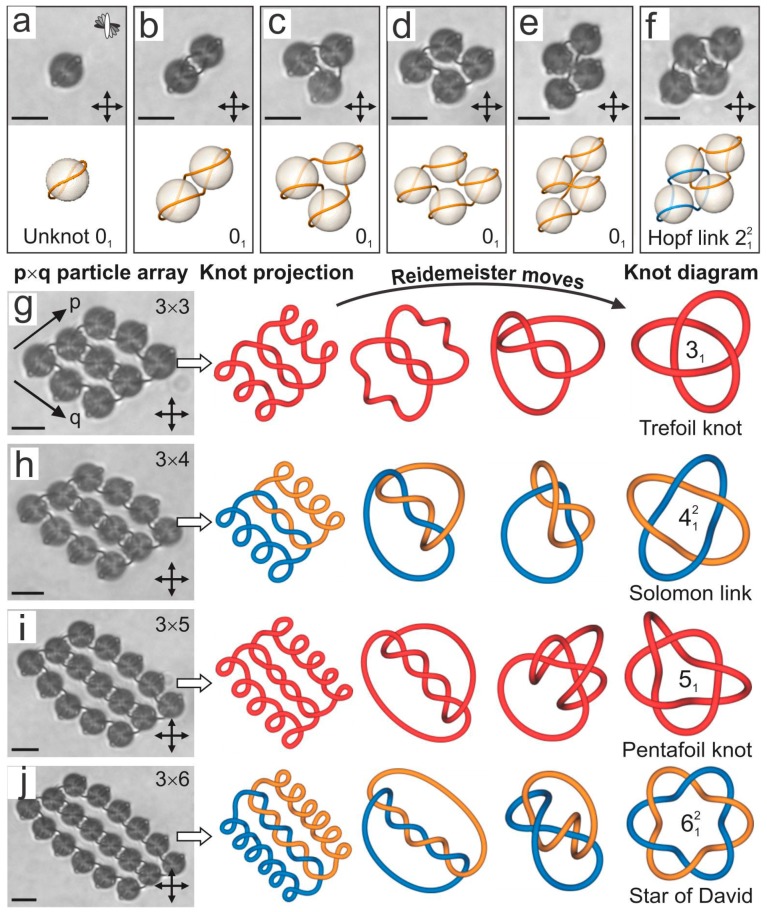
Defect loops tie links and knots in chiral nematic colloids. (**a**) A twisted Saturn ring is an unknot and appears spontaneously around a microsphere in a chiral nematic. The molecular arrangement in all the panels is illustrated in the top-right corner. (**b**–**e**) The defect loops of the colloidal dimer, trimer and tetramers are also unknots. (**f**) The first non-trivial topological binding of a colloidal cluster is the Hopf link. Two separate defect loops are interlinked. In the lower panels (**a**–**f**) the loop conformations were calculated using numerical Landau-de Gennes modeling. (**g**–**j**) A series of alternating torus knots and links on colloidal particle arrays of m × n particles are knitted by the laser. The actual defect loops are schematically redrawn to show the relaxation mapping from the experimental planar projection to the final knot diagram. The knots are labeled using the standard notation C, i, N, where C is the minimum number of crossings, i distinguishes the different knot types and N counts the number of loops. Scale bars, 5 µm. Adapted from Ref. [87].

When the entanglement experiments are performed in a chiral nematic liquid crystal, another class of entanglement is immediately observed, which is illustrated in Figure 14 [87,88,89].

When the number of colloidal particles is small (up to 4), we observe a trivial topological loop conformation, consisting of a single loop encircling a single colloidal particle (Figure 14a) or entangling a colloidal dimer (Figure 14b), trimer (Figure 14c) or tetramer (Figure 14d,e). Here, the chiral environment is simply a 90° left-twisted nematic cell, with the molecular arrangement represented in the upper-right corner of Figure 14a. All the images are taken between crossed-polarizers and the field of view is bright because of the waveguiding of the polarization in a twisted nematic structure.

The first topologically nontrivial object is observed in Figure 14f, where a colloidal tetramer is entangled by two separate, mutually interlinked loops. The number of loops and their configuration in space is determined experimentally by changing the focus of the microscope and following the trajectories of the loops, which always appear dark because of the scattered light. In this way it is possible to reconstruct a planar projection (i.e., the projection on a microscope plane) for practically any kind of entangled loop. The colloidal clusters in Figure 14a–f were built using laser tweezers that enable grabbing and then manipulating the individual colloidal particles into a preferred colloidal constellation [89,90]. Once a selected number of particles is brought together, the laser tweezers are used to cut and rewire the individual defect loops in many possible configurations.

In the continuation, the colloidal array was enlarged by systematically adding rows of particles to the pre-existing p × q particle array, as shown in Figure 14g–j. At the same time, the laser tweezers were used to systematically cut and rewire the defect loops, as shown in the sequence of microscope images in Figure 14g–j. For example, Figure 14g shows nine colloidal particles arranged in a 3 × 3 particle array. There are many possible methods of loop formation and entanglement in this colloidal array. However, for the sake of a systematic study, the individual loops of colloidal particles were manipulated in a way that results in a single defect loop running all around the colloidal particles and entangling them in a firmly bound structure. The question that poses itself is: What is the topology of this loop? To answer this question we graphically extract the loop and form the projection of the loop on the imaging plane, which is shown with the first red line labeled “knot projection” in Figure 14g. It is immediately clear that there is a single defect loop and its topology is elucidated by applying Reidemeister moves [90]. These moves make the knot projection smoother and do not change the topology of the object. Finally, we obtain the knot diagram of this loop, which turns out to be a Trefoil knot. It has three crossings and a single loop. 

By adding an additional three colloidal particles to the 3 × 3 particle array and manipulating the defect loops to continue the entanglement in a logical way, we obtain an entangled colloidal array of 3 × 4 colloidal particles. It turns out that this is the Solomon link, consisting of two loops that are mutually interlinked to form four crossings. Note the similarity of the Solomon link with four crossings and the Hopf link with two crossings in Figure 14f. Using the procedure of adding particles in a logical way, we obtain 3 × 5 particle array that is entangled with a Pentafoil knot, as shown in Figure 14i. By adding an additional three particles we end up with a colloidal array that is entangled by a Star of David. We see from Figure 14 that the systematic addition and building of colloidal arrays results in a series of alternating torus knots and links. This result indicates that a generically knotted series of knots and links is possible and that knots and links of arbitrary complexity can be formed by taking a sufficiently large colloidal array.

**Figure 15:**
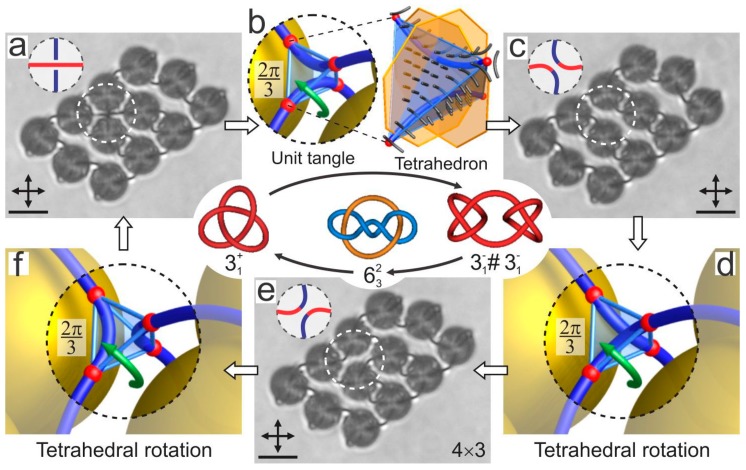
Rewiring of knots and links using laser tweezers. (**a**) A right-handled Trefoil knot is realized on a 4 × 3 colloidal array. The dashed circles indicate a unit tangle that can be rewired with the laser beam. The tangle consists of two perpendicular line segments and the surrounding molecular field. (**b**) By rewiring the unit tangle that corresponds to a 2*π*/3 rotation of the encircled tetrahedron, a new composite knot, shown in (**c**), is knitted. The sequence of tangle rewirings in (**b**–**f**) results in switching between the knots and links, demonstrated in (**a**,**c**,**e**). The procedure for rewiring the topological defect loops is not an exact and repeatable procedure, but is rather a skill that is learned after trying and repeating a good number of experiments. It is necessary to learn how to adjust the applied power, the position of the tweezers’ focus and the direction of the movement of the trap that is cutting the defect loop. Scale bars, 5 µm. Adapted from Ref. [87].

The knots and links of topological defect loops entangling colloids in chiral nematics can also be reversibly retied. This involves using the strong light of the laser tweezers to locally influence the defect loops in order to rewire them into another configuration. Certain topological rules apply when such a rewiring is performed, as illustrated in Figure 15. The first panel in Figure 15a shows an array of 4 × 3 colloidal particles, and we are concentrated on the possible rewiring of the tangled crossing indicated by the white dashed circle in Figure 15a. The local constellation of the two defect lines that are crossing each other in this area is shown in the upper-left corner of this panel. If we enlarge this panel, as in Figure 15b, we see a graphical visualization of a small volume in between the four colloidal particles where the line crossing takes place. An interesting feature is observed: It is possible to construct a small tetrahedron with the corners illustrated by red dots in Figure 15b. The corresponding illustration of the director field inside this tetrahedron shows that it has a certain symmetry. Namely, the tetrahedron could be rotated by steps of 120° around a symmetry axis of the tetrahedron that is running through its corner. By performing a 120° rotation (anti-clockwise green arrow in Figure 15b) the director field in the tetrahedron is preserved, but the two defect lines are now rewired, as shown in the next panel in Figure 15c. This imaginary rotation of the tetrahedron, which is allowed by local symmetry, results in the rewiring of the crossings and changes the global topology of the colloidal cluster. We can make exactly three possible tangles by reversibly transforming one into another. These local transformations change the topology and the handedness of the knots and links [91].

In real experiments this colloidal cluster is then systematically knotted and linked with the laser tweezers into the desired configurations by a localized laser-induced micro-quench. This is achieved by adjusting the laser power to the level that influences locally the defect loops and enables their rewiring. In the example given in Figure 15 we start with the right-handed Trefoil knot (Figure 15a), which is transformed in the left-handed composite knot in Figure 15c and, finally, into the third possible configuration, which is the two-component link in Figure 15e. It turns out that practically any kind of knot or link can be realized by taking a sufficiently large colloidal array. 

## 6. Exotic Nematic Colloids: Handlebodies and Koch-Star Particles 

So far we have presented the rich topology of a nematic liquid crystal around a spherical or fiber-like inclusion, i.e., all topologically trivial objects. These experiments clearly demonstrate that the main parameter that enhances the richness of the observed topological structures is the chirality of the nematic liquid crystal. For achiral liquid crystals the topological defects around colloidal particles are rather simple, with the most complex being the colloidal entanglement of a pair or several particles. However, when the nematic liquid crystal is made chiral, the topology of the nematic director becomes extremely rich, as demonstrated by the knotting and linking of the nematic orientational field around multiple spherical colloids. Nevertheless, the topological properties of these inclusions are rather trivial since microspheres are all topological objects with genus g = 0, i.e., having no handles.

Modern micro-fabrication techniques allow us to fabricate colloidal particles with complex shapes, such as tori and other handlebodies [70], spiraling microrods [92], Koch-star microparticles [93], and even knotted and linked polymer colloidal particles [94]. It is possible to produce such particles by using 2D lithography or 3D two-photon photo-polymerization techniques. The ability to produce topologically nontrivial colloidal particles raises the question of the resulting topology of the director field that is forced to align along the closed surfaces of these colloidal particles.

The topology of the director field around the colloidal particles is governed by the law of the conservation of topological charge [2]. This law is similar to the law of the conservation of electric charge, but instead of the electric field, we have the director field, which describes the ordering of the nematic liquid crystal. Topological charges in nematic liquid crystals are therefore associated with singularities of the director field, which appear in the core of point defects, also called hedgehogs, and the defect lines or loops, also called disclination lines. The law of the conservation of topological charge is similar to Gauss’s law for the electrostatic field, and the topological charge is measured in a similar way. To measure the topological charge, a closed surface is constructed around the field’s singularity and the topological flux is measured by integrating it along the closed surface. The resulting topological charge q is formally calculated as an integral of the topological flux (i.e., the number of director “streamlines”) along the closed surface embracing this singularity:

(1) $$q=\frac{1}{8\pi }\oint_{\sigma }\varepsilon_{ijk}\vec{n}\cdot \left(\frac{\partial \vec{n}}{\partial x_{j}}\times \frac{\partial \vec{n}}{\partial x_{k}}\right)dS_{i}$$

Here, $\varepsilon_{ijk}$ is the Levi-Civita totally asymmetric tensor and $x_{i}$ are the Cartesian coordinates, and $dS_{i}$ is the surface differential. This expression for the topological charge is odd in the director field $\vec{n}$ and, as a consequence, the sign of the topological charge for our director field is not well defined. The nematic director is a “headless” vectorial field where +**n** and −**n** are formally equivalent. The expression “headless” actually tells us that we are using an inappropriate vectorial field for the description of the tensorial field. The director field is a tensorial field and the direction (and the director) defines the direction of the long axis of the molecular probability distribution. As a consequence, the sign of the topological charge in the nematic liquid crystals has no meaning, the only strict condition is that there are two kinds of charges and they are different in signs. This difference in signs helps us to count the total charge, which in many cases mutually compensates. Whereas in vectorial fields the combined topological charge for two hedgehogs is simply the algebraic sum of their charges, in nematics, where the director field is tensorial, the combined charge of two hedgehogs is either the sum or the difference of their charges.

In nematic colloids, solid, liquid or gas particles are inserted into the nematic liquid crystal and the nematic liquid-crystal molecules are forced to align along the closed surface of the inclusions. There are two characteristic types of local alignment for the liquid-crystal molecules at the interface: Parallel and perpendicular. In rare cases, the surface alignment is oblique. Because the molecules are forced to align all along the closed surface, topological defects have to be created in the surrounding nematic liquid crystal, as in general it is not possible to fill the surrounding orientational field without orientational singularities, which are our topological defects.

It turns out that the topological charge of the surrounding defects created by the immersion of the colloidal particles into the nematic liquid crystal is determined by a topological invariant called the Euler characteristic χ of the inserted particle. The Euler characteristic is in a one-to-one correspondence with the topology of the immersed particle and does not change under any smooth transformation of the surface of the particle. The Euler characteristic for a closed surface depends on the type of the closed surface and is directly related to the number of handles and holes that the object possesses. For example, a sphere has a closed surface with no handles and holes and its Euler characteristic is equal to χ=2. If we make one hole through the sphere, we obtain a torus with a different Euler characteristic of χ=0. More precisely, the Gauss-Bonnet theorem claims that the Euler characteristic χ of any closed surface is related to the total Gaussian curvature of that surface:

(2) $$\chi =\frac{1}{2\pi }\oint_{\sigma }KdS=\oint_{\sigma }d\theta \;d\Phi \;\vec{\nu }\cdot \left[\frac{\partial \vec{\nu }}{\partial \theta }\times \frac{\partial \vec{\nu }}{\partial \Phi }\right]$$

Here, $K=\kappa_{1}\cdot \kappa_{2}$ is the local Gaussian curvature and $\kappa_{i}$ are the two principal curvatures at a given point on the surface. The vector $\vec{\nu }$ is the local normal to the surface σ at the point of consideration, defined by the two angles θ and Φ. Furthermore, it can be shown that the Euler characteristic is related to the genus g of the surface of the particle:

(3) χ=2(1−g)

Here, the genus g is the number of handles attached to the sphere that results in the formation of handle-bodies. For example, we obtain a torus by adding a single handle to the sphere and then smoothly transforming this closed surface into a torus.

If we now consider any closed surface inserted into a vector field when this field is perpendicular at all the points of the inserted surface, the topological charge q of this surface is equal to the integral given by Equation (1). This surface integral reduces to the total Gaussian curvature of the closed surface divided by 4π. Because the Gauss-Bonnet theorem states that the total Gaussian curvature of any closed surface without a boundary is quantized in units of 2π (Equation (2)), the resulting hedgehog charge $m_{c}$ of the vectorial field aligned along the surface *S* is:

(4) $$m_{c}=\pm \frac{2\pi \cdot \chi }{4\pi }=\pm \left(1-g\right)$$

Far away from the surface, the field is considered homogeneous and the topological charge of this larger volume is zero. On the other hand, there must be topological defects inside this volume, carrying the topological charge $m_{d}$, which compensates for the topological charge of the surface:

(5) $$m_{c}+m_{d}=0$$

For a vector field the Euler characteristic of the immersed surface of the particle therefore uniquely defines the total topological charge $m_{d}$ of all the defects accompanying the surface:

(6) $$m_{d}=1-g$$

For a sphere the genus g=0, which means that the net charge of all topological defects accompanying the sphere should add up to $m_{d}=1$. In principle it is, therefore, allowed to have many different topological defects accompanying the sphere, which mutually compensate for their charge, except for a single hedgehog charge $m_{d}=1$. However, all these additional charges increase the elastic energy and in reality in most cases we observe a single hedgehog unit charge accompanying the sphere. This is in perfect agreement with the observation of a point hedgehog or a Saturn ring encircling the microspheres in the nematic liquid crystals. Moving from spheres with g=0 to tori with g=1, the theorems require that the total topological charge of the hedgehogs accompanying the torus should be zero, $m_{d}=0$. There are two possibilities in the case of a torus: (i) there are no topological defects accompanying the torus or, (ii) there is an even number of topological defects that are oppositely charged and mutually compensate. 

Both scenarios were observed for a torus [95,96,97,98]. The first case with no topological singularities was observed in the experiment where nematic toroidal dispersions were created [95,96]. In this case, the torus is filled with the nematic that creates a planar boundary condition that requires no topological defects inside the torus. The second scenario of mutually compensating pairs of topological defects floating outside the solid micro-torus and handlebodies was observed by Senyuk et al. [70], Campbell et al. [97] and Tasinkevych et al. [98]. By using fluorescent confocal microscopy they were able to reconstruct a director field around the particle and they observed that the topological defects were oppositely charged, thus preserving the total topological charge of zero.

**Figure 16:**
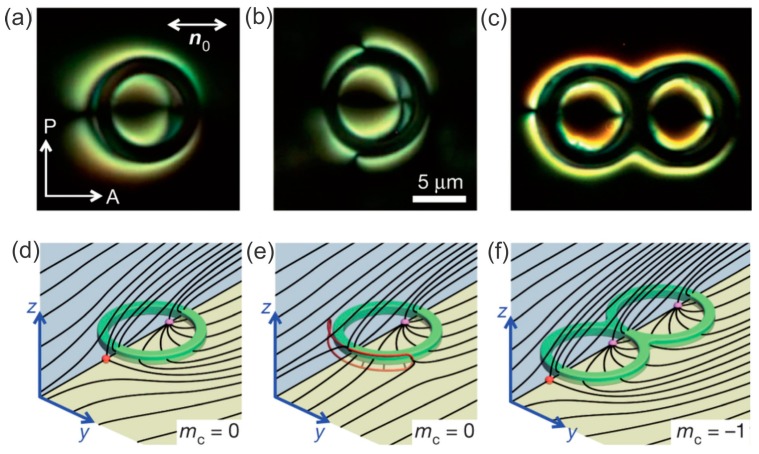
(**a**,**b**) Cross-polarized images of a toroidal particle with perpendicular surface anchoring of the liquid-crystal molecules immersed in a planar nematic cell. Two point defects can be seen in panel (**a**). In panel (**b**) one point defect has opened into a small loop. (**c**) A handlebody with genus g=2 is accompanied by three point defects. (**d**–**f**) Schematic drawing of the director field for the three images in (**a**–**c**). The scale bar is 5 µm. Adapted from Ref. [70].

Figure 16a,b show a toroidal colloidal particle made of photo-polymerizable material with a perpendicular surface anchoring immersed in a planar nematic liquid-crystal cell [70]. It is clear that there are two topological defects: One centered inside the toroidal particle and the other resting on its outer perimeter. In Figure 16b the point defect has opened into a small loop. The panels in Figure 16d,e show the schematics of the director field around a toroidal particle with perpendicular surface anchoring of the liquid-crystal molecules.

**Figure 17:**
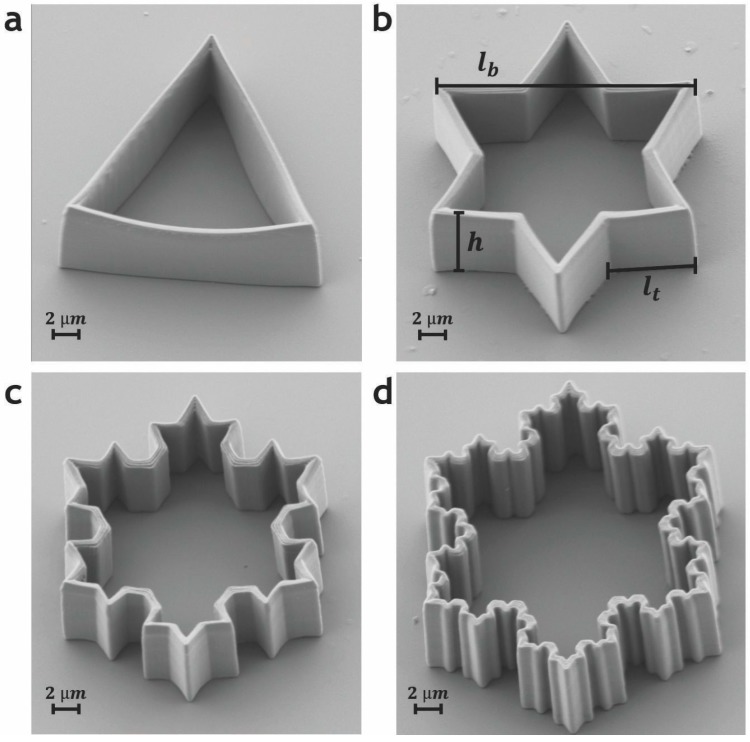
Scanning electron images of Koch-star colloidal particles produced by a two-photon polymerization technique using the Photonic Professional System by Nanoscribe. Note that these particles are topologically equivalent to torii with g = 1. Adapted from Ref. [93].

A smooth torus can be morphed into a Koch-star colloidal particle that is produced mathematically following the prescribed procedure for constructing the particles. Bending and shaping the torus keeps its Euler characteristic unchanged, only the geometry and shape are changing. This means that the Koch-star particles, shown in Figure 17, are topologically equivalent to a torus and the total charge of all defects of a Koch-star particle should always be zero.

**Figure 18:**
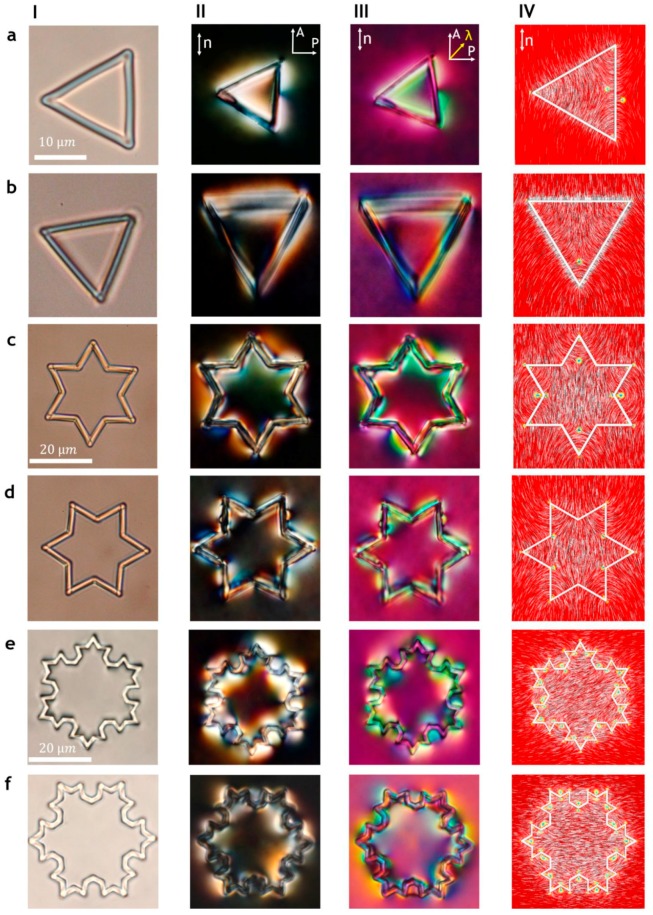
Koch-star colloidal particles of iterations 0 (**a**,**b**), 1 (**c**,**d**), 2 (**e**) and 3 (**f**) with a fractal pattern of the director field reveal nematic topological states. Panels **I** are taken with no polarizers; panels **II** are taken between crossed polarizers; panels (**III**) are taken between crossed polarizers and the red plate; panels IV are numerical Landau de Gennes simulations. Adapted from Ref. [93].

The topological equivalence of Koch-star particles and toroidal particles is demonstrated in the examination of the number of topological defects around such particles with polarized optical microscopy and laser tweezers [93]. Figure 18 shows some examples of Koch-star particles having different iterations as viewed under an optical microscope. Panel I shows the particle in the isotropic phase of the liquid crystal, where the optical irregularities due to production can be detected. Panel II shows the crossed-polarized images of Koch-star particles in a uniformly aligned planar nematic cell, where these particles are levitating in the middle of the nematic layer. If there was no surface alignment of the liquid crystal on these particles, the crossed-polarized images would be completely black. There is a strong surface alignment in the direction perpendicular to the local surface, which means that the orientation of the liquid crystal is strongly distorted in the vicinity of the polymer walls of the Koch-star particles. Because of this rather large distortion, extending micrometers from the surfaces, the direction of the local optical axis is no longer parallel to the far-field liquid-crystal alignment. This local rotation of the optical axis results in strong birefringent colors accompanying the walls of the Koch-star particles. These colors are not uniform along all the sides of the Koch-star particles. For example, for the first iteration Koch-star particle (a triangle), shown in Figure 18 IIa, we can clearly see uneven coloring of the liquid crystal along the side parallel to the far-field director. This indicated the presence of a topological defect in the middle of this side of the triangle. Additional optical inhomogeneities are clearly observable in the corner of the triangle, where topological defects are also located. Similar color inhomogeneities are observed in panels III, where a special optical polarizing technique is used (the red plate or the *λ* plate), which helps us to determine the local alignment of the optical axis of the liquid crystal. In combination with optical tweezers, these optical polarization techniques allow us to count the number of topological defects. 

When we consider the next iterations of the Koch-particles, the optical pattern is even more complicated, as shown for the Star of David particles in Figure 18c. Nevertheless, the number of topological defects can be counted, and it turns out that there is always an even number of topological defects that mutually compensate and result in the overall zero topological charge. The experimental observations were complemented by numerical simulation using the finite-element method, and the resulting director fields together with topological defects are shown in panel IV. The number of topological defect pairs increases exponentially with fractal iterations and equals 108 at the third fractal iteration.

There are other examples of colloidal particles of exotic shapes in liquid crystals, such as spiraling rods, prisms, platelets, knots and links, etc. They all show topological defects that obey the fundamental law of the conservation of topological charges, which makes liquid crystals a unique playground for the topology of fields.

## 7. Conclusions

This short review presents aspects of the rapidly developing subfield of liquid-crystal colloids, which has generated many attractive and fascinating results over the past 10–15 years. Of course, it is not possible to present all the important achievements in this field; this would be a task comparable to writing a book. Instead I have presented a rather personal perspective with an emphasis on experimental topology. Indeed, the experimental topology involving studies of topological defects, their structure, emergence and dynamics is in my opinion one of the most appealing phenomena in nematic colloids. Defects in liquid-crystal colloids are relatively easy to observe, study and manipulate, thus giving us an insight into the foundations of condensed-matter topology. This fundamental aspect of nematic colloidal science is complemented by emerging applications of topological defects in future optical and photonic devices. One of them is generating optical wave fronts with complex structures, such as Laguerre Gaussian beams, where the topological charge of the condensed matter is imprinted into the topological charge of the electromagnetic waves.

## 8. Materials and Methods

### 8.1. Preparation of Nematic Colloids

The simplest way to prepare nematic colloids is by mixing the microspheres of different materials in a nematic liquid crystal. Typically, the nematic liquid crystal 5CB is used in all experiments because it is commercially available, stable and its properties are well known. The typical material for the microspheres is silica, because of its excellent surface smoothness, ease of surface functionalization and commercial availability over a large range of diameters. One of the most important aspects of colloidal preparation is the chemical treatment of silica microspheres to induce excellent surface alignment. A good way to obtain the surface anchoring is to treat the silica microspheres with silanes such as DMOAP. This surface modification creates a well-defined and stable monolayer of DMOAP molecules, which aligns practically all liquid crystals perpendicular to the surface. The method of surface preparation is described in detail in Refs. [2,21,25].

The next step is the introduction of dried and surface-functionalized silica microspheres into a nematic liquid crystal, typically with a weight ratio of 1%. After mixing the colloidal mixture, the micrometer colloidal particles are usually well dispersed. However, smaller nematic colloids tend to preserve the clusters and aggregates of particles, and have to be treated with strong ultrasound to break up these clusters. After the mixture is prepared it is introduced by capillary action into a measuring cell. This is a standard cell made of two parallel glass plates separated by a spacer, which defines the thickness of the gap to be filled with the nematic colloidal dispersion. The cell is pre-treated in order to fabricate an alignment layer, which then aligns the director of the nematic liquid crystal uniformly throughout the cell surfaces. This alignment can be either parallel (planar cell) or perpendicular (homeotropic cell) to the surface of the cell walls.

### 8.2. Fabrication of Colloidal Particles Using 2-Photon Polymerization

Whereas particles with a spherical or rod-like shape are commercially available, micrometer-sized particles of practically arbitrary shape can be produced using the 2-photon polymerization of a light-sensitive polymer. Typically, commercially available instruments, such as Photonic professional (Nanoscribe GmbH, Eggenstein-Leopoldshafen, Germany), are able to produce tens of micrometer-sized particles with complex shapes using different photosensitive polymers. These particles are produced on a supporting glass surface and have to be surface functionalized, detached from the glass surface and introduced into the liquid crystal. DMOAP is used to ensure a good homeotropic surface anchoring of the liquid crystal on a typical 2-photon polymer. However, it turns out that not all photosensitive polymers can be functionalized with DMOAP.

### 8.3. Manipulation of Nematic Colloids by Laser Tweezers

Once the nematic colloidal dispersion is introduced into the cell, the particles can only be moved and manipulated with a non-contact method, such as laser tweezers. These optical tweezers are a rather simple instrument, consisting of a strong laser source, a microscope and the system for moving the focus of the laser beam in the microscope. Typically, a 100-mW laser is used (such as CW Nd:YAG), which is sent to one of the entrance ports of an inverted optical microscope. The laser light is then focused in the focal plane of the objective. When the measuring cell with the nematic colloidal dispersion is observed in such a microscope, the laser focus is in the same imaging plane as the observer (or camera). At a low laser power (several mW) the optical trap has a relatively small effect on the colloidal particles. In this regime, the refractive index of the colloidal particles plays an important role. It is known from optical trapping that colloidal particles can be trapped by the tweezers only if their refractive index is larger than the index of the surroundings. This is difficult to achieve in liquid crystals, since the refractive indices of typical liquid crystals are between 1.5 and 1.7. This is close to or even higher than the refractive index of glass (1.5), which means that glass particles in a nematic liquid crystal cannot be trapped and manipulated by the tweezers. However, it was shown in 2004 that the optical trapping of particles in liquid crystals is much more complex than the optical trapping of particles in ordinary liquids. There are two reasons for this: (i) optical trapping in a liquid crystal is polarization sensitive, which means that the electric field of the laser light couples to the dielectric anisotropy of the liquid crystal. Close to the colloidal particles, the liquid crystal is aligned along the normal to the surface of the particle, which creates an elastically distorted region around the particle. The polarized light of the tweezers couples to that region and by moving the trap, we also move the region. The particle is forced to follow the trap, because it has to follow the moving, elastically distorted region around itself. (ii) If the power of the laser tweezers is strong (100 mW), the liquid crystal is locally heated by the absorption of light. This creates a small volume of liquid crystal where the order parameter and the elastic properties are different from the surroundings. Such a volume acts as an artificial particle, which attracts other colloidal particles. By using either of the two mechanisms we can trap and manipulate colloidal particles of an arbitrary material and shape in the nematic liquid crystal.
